# D’or: deep orienter of protein–protein interaction networks

**DOI:** 10.1093/bioinformatics/btae355

**Published:** 2024-06-11

**Authors:** Daniel Pirak, Roded Sharan

**Affiliations:** Department of Electrical Engineering, Tel Aviv University, Tel Aviv 69978, Israel; Department of Computer Science, Tel Aviv University, Tel Aviv 69978, Israel

## Abstract

**Motivation:**

Protein–protein interactions (PPIs) provide the skeleton for signal transduction in the cell. Current PPI measurement techniques do not provide information on their directionality which is critical for elucidating signaling pathways. To date, there are hundreds of thousands of known PPIs in public databases, yet only a small fraction of them have an assigned direction. This information gap calls for computational approaches for inferring the directionality of PPIs, aka *network orientation*.

**Results:**

In this work, we propose a novel deep learning approach for PPI network orientation. Our method first generates a set of proximity scores between a protein interaction and sets of cause and effect proteins using a network propagation procedure. Each of these score sets is fed, one at a time, to a deep set encoder whose outputs are used as features for predicting the interaction’s orientation. On a comprehensive dataset of oriented PPIs taken from five different sources, we achieve an area under the precision–recall curve of 0.89–0.92, outperforming previous methods. We further demonstrate the utility of the oriented network in prioritizing cancer driver genes and disease genes.

**Availability and implementation:**

D’or is implemented in Python and is publicly available at https://github.com/pirakd/DeepOrienter.

## 1 Introduction

Protein–protein interactions (PPIs) provide the skeleton for signal transduction in the cell. However, current high-throughput PPI measurement techniques do not provide information on the direction of interactions. This type of information has been shown to be instrumental in various biological tasks such as discovery and reconstruction of signaling pathways in yeast (Gitter *et al.* 2010), retrieval of unknown pathway modulators in human (Vinayagam *et al.* 2011), drug design (Csermely *et al.* 2005), and prediction of proteins function (Cao *et al.* 2014). While not all interactions are necessarily directed, prior research predicted that about 2/3 of them are Silberberg *et al.* (2014). Thus, there is a growing need to infer interaction directions computationally (Pandey and Loscalzo 2023), a task known as *network orientation*.

Earlier works in this area mostly used unsupervised techniques to infer edge directions. These techniques relied on pairs of cause and effect proteins derived from perturbation experiments together with the notion that there must be a path in the network between the two for the effect to take place. A major challenge in this approach is the complexity of considering all possible paths between two nodes. Yeang *et al.* (2004) used a probabilistic model together with a max-product algorithm to infer orientation and sign of edges but their solution was limited to very small networks with short connecting paths (Gitter *et al.* 2010). Later, Gitter *et al.* (2010) used random orientation of edges followed by a greedy local search to connect known endpoints of signaling pathways with paths of bounded length. Silverbush and Sharan (2014) overcame path length limitation by formulating an integer program which considers only the shortest path between any cause–effect pair. A caveat of this method is its low recall as most edges do not lie on short paths from causes to effects.

Previous supervised approaches are scarcer. One approach by Vinayagam *et al.* (2011) used shortest path statistics between membrane receptors and transcription factors to train a Naïve Bayes classifier for predicting edge directions. However, limited edge coverage by shortest paths hampered this approach. Another approach, D2D, by Silverbush and Sharan (2019) classified edges based on the assumption that heads and tails of oriented edges should have higher proximity to cause and effect protein sets, respectively. As the method is applicable to any edge, it overcame the recall problem of previous approaches producing state-of-the-art results for the problem. Yet, D2D is based on heuristic approaches for evaluating set-to-node proximity, according to the sum of proximities of each member in the set, as well as on arbitrary choices of how to compare proximities to causes and effects. These choices as we show below greatly affect the method’s performance.

In this work we present D’or, the first deep learning based method for orienting PPIs. D’or uses sets (or distributions) of proximity scores from available cause–effect pairs as input to a deep learning encoder, which is trained in a supervised fashion to generate features for orientation prediction. A key novelty of D’or is its ability to learn a general function of proximity scores rather than using arbitrary measures such as a sum, used by D2D to aggregate node scores, or a ratio, used by D2D to contrast causes with effects. On a comprehensive dataset of oriented edges taken from five different sources, we achieve an area under the precision–recall curve of 0.89–0.92, outperforming previous methods. We further demonstrate that the oriented network can aid in prioritizing disease-associated genes and in particular cancer driver genes.

## 2 Materials and methods

### 2.1 The D’or algorithm

**Figure 1.**
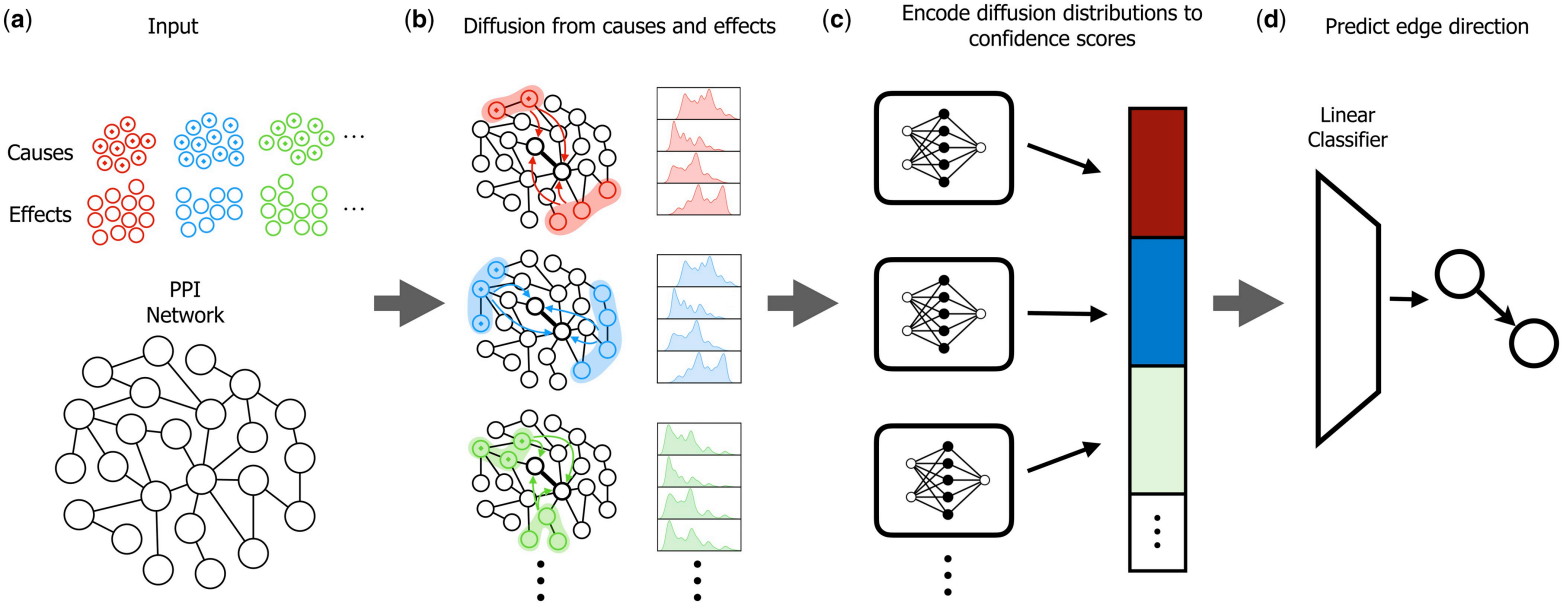
An overview of D’or. (a) The algorithm receives as input a list of cause–effect pairs of gene sets and an undirected PPI network. (b) Information is diffused from the cause and effect vertices sets to a pair of vertices incident to an edge of interest. This results in four distributions of diffusion scores (one for each node-set couple). (c) Each distribution quartet is encoded to a scalar feature that represents the confidence in an edge direction. (d) A linear classifier assigns a final confidence score to the edge’s orientation.

We devised an orientation algorithm called Deep Orienter (D’or). D’or learns functions over sets of propagation scores from cause and effect genes to a target PPI in order to predict an orientation for that interaction. The algorithm consists of three main parts (Fig. 1): (i) computation of network propagation scores from cause and effect gene sets to genes that are incident to the target interaction. (ii) A deep learning model that encodes these scores into set-function-based features for each cause–effect set pair. And (iii) linear classification of the resulting features followed by a softmax function to produce the final prediction scores. We describe these components in detail below.

**Network propagation.** Our starting point is the computation of proximity scores of each vertex to given sets of causal or effect proteins. These proximity scores are given by solutions to the following system of equations:

(1) $${\left\{F(v)=\alpha \left[\sum_{u\in N(v)}F(u)w(u,v)\right]+(1-\alpha )P(v)\right\}}_{v\in G}$$

where *P*(*v*) is called the prior term for vertex *v* and is set to 1 for input cause or effect proteins and 0 otherwise. *α* is a smoothing parameter that balances between the network and the prior term and is set to 0.8 following the previous work (Silverbush and Sharan 2019). *w* is a normalized edge weight matrix obtained by $W=D^{-1/2}AD^{-1/2}$, where *A* is the network’s adjacency matrix and *D* is a diagonal matrix of weighted degrees. For partially directed networks, the symmetric normalization above does not distinguish between in-going and out-going edges, hence we normalize by $W=AD^{-1}$. To ensure convergence of the subsequent diffusion process, the network must be connected in the undirected case and strongly connected otherwise.

**Figure 2.**
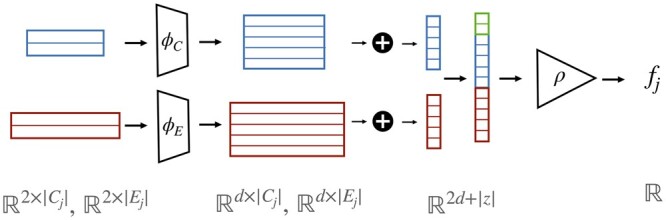
A single D’or block. Propagation scores from the causal set (blue) and effect set (red) are used as inputs to two neural networks ($\phi_{C},\;\phi_{E}$), whose outputs are being averaged separately and then concatenated together with an addition of the block’s index embedding. This concatenated vector is used as an input for another neural network (*ρ*).

**Constructing set-function-based features.** Unlike previous works such as D2D or the method of Vinayagam *et al.*, which use hand-crafted features for orientation prediction, D’or aims to learn a more general family of set functions, avoiding potential information loss. For a single pair of cause and effect protein sets *S_j_* and *E_j_* and a target interaction (*u*, *v*), D’or assigns a confidence measure to each of its possible orientations by:

(2) $$\begin{array}{c} \mathrm{scor}e_{j}(u\to v)=\\ \rho (\left[\frac{1}{\left|C_{j}\right|}\sum_{c_{i}\in C_{j}}\phi_{C}(F^{c_{i}}\left(u\right),F^{c_{i}}\left(v\right))\right]\;||\\ \left[\frac{1}{\left|E_{j}\right|}\sum_{e_{i}\in E_{j}}\phi_{E}(F^{e_{i}}(u),F^{e_{i}}\left(v\right))\right]\;||\;emb_{j}) \end{array}$$

where $F^{c}(u)$ stands for the diffusion score of node *u* when propagating from node *c*. $\phi_{C}$ and $\phi_{E}$ are two fully connected neural networks operating on each element of the set separately. *emb_j_* is a learned embedding vector, of dimension *z* and unique for each set pair (*C_j_*, *E_j_*). *ρ* is another fully connected neural network. The model is summarized in Fig. 2.

**Learning set-functions.** The network structure borrows ideas from the DeepSets framework (Zaheer *et al.* 2017) that learns set functions, i.e. functions that are invariant to element permutations and can be applied to inputs of varying size. It was shown that for countable sets, all set-functions can be represented as $\rho \left(\sum_{x\in X}\phi \left(x\right)\right)$, where *X* is a set and ϕ and *ρ* are some suitable transformations. DeepSets approximates ϕ and *ρ* using neural networks.

Similarly, in D’or we aim to learn a function over two sets with a model of the following form: $f(X_{1},X_{2})=\rho \left(\sum_{x\in X_{1}}\phi_{1}(x),\;\sum_{x\in X_{2}}\phi_{2}(x)\right)$. One can generalize the proof in (Zaheer *et al.* 2017) to show that such a form captures all functions on pairs of sets:

Theorem 1. *Let* $X_{1}=\{x_{1}^{1},\ldots ,x_{M}^{1}\}$*and*$X_{2}=\{x_{1}^{2},\ldots ,x_{N}^{2}\},\;x_{i}^{1},x_{j}^{2}\in \Gamma ,\;\forall i,j\;$*be two sets whose elements are countable. A function*$f(X_{1},X_{2}):\;2^{\Gamma }\times 2^{\Gamma }\to R$*is permutation invariant for each set separately iff it can be decomposed to* $\rho \left(\sum_{x\in X_{1}}\phi_{1}(x),\;\sum_{x\in X_{2}}\phi_{2}(x)\right)$*for suitable* $\phi_{1},\phi_{2},\rho$.

Proof. The “if” part follows by observing that the separate summation of the elements of the two sets implies that the order of their elements becomes irrelevant. Conversely, since Γ is countable there is a mapping c:Γ→N. By choosing $\phi_{1}=\phi_{2}=4^{-c(x)}$ we get that $\sum_{x\in X_{1}}\phi_{1}(x)$ and $\sum_{x\in X_{2}}\phi_{2}(x)$ form a unique mapping each of the sets $X_{1},X_{2}\in \Gamma$, hence the pair $\left(\sum_{x\in X_{1}}\phi_{1}(x),\;\sum_{x\in X_{2}}\phi_{2}(x)\right)$ forms a unique mapping for any combination of the sets above. It follows that a function $\rho :R^{2}\to R$ can be constructed such that $f(X_{1},X_{2})=\rho \left(\sum_{x\in X_{1}}\phi_{1}\left(x\right),\;\sum_{x\in X_{2}}\phi_{2}\left(x\right)\right)$. □

**Classification and loss function.** The complete model consists of applying the D’or block (Fig. 2) on multiple cause–effect pairs. The block’s weights are shared across pairs except of the embedding vector that is unique to each pair. Finally, the *N* outputs are fed into a linear layer with a softmax activation function which outputs a final prediction for the edge (u, v). The objective function for D’or includes two parts. The first part is the binary cross-entropy (BCE) between the classifier’s outputs and the true labels. The second part is a regularization term which sums the BCE between the output of each block and the true label. The two parts are combined as follows:

(3) $$\begin{array}{c} \mathrm{Loss}(u\to v)=\beta \cdot \text{BCE}(f(u\to v),\;y)+\\ \left(1-\beta )\cdot \frac{1}{N}\sum_{j=1}^{N}\text{BCE}(\mathrm{scor}e_{j}(u\to v),\;y),\;\beta \in [0,1\right] \end{array}$$

where *y* is the true direction, f(u→v) is the output of the final linear layer for the orientation $\left(u\to v),\;\mathrm{scor}e_{j}(u\to v\right)$ is the output of the *j*th block and *β* is a model parameter. Equation (3) represents the loss term of a single prediction, the total loss is calculated as an average over all predictions losses.

### 2.2 Consensus orientation

For each possible edge orientation (u→v) and each model *m*, we assign a direction certainty measure in the form of the probability ratio $f_{m}(u\to v)/f_{m}(v\to u)$. The consensus score of several models is given by the sum of log-likelihood of these ratios:

(4) $$S_{\text{consensus}}(u\to v)=\sum_{m\in M}\;\text{log}\;\frac{f_{m}(u\to v)}{f_{m}(v\to u)}$$

where *M* is the group of models. Finally, we say that the edge (*u*, *v*) is oriented from *u* to *v* if $S_{\text{consensus}}(u\to v)$ lies in the top *q* percentile of scores. For evaluations including hard predictions, we chose *q* such that 80% of the edges are oriented.

### 2.3 Implementation details

**Model optimization and training process.** For hyperparameter optimization we performed a simple random search, in which we trained hundreds of models with different configurations over the training data. The configuration of the best performing model was then chosen for evaluation. We optimized for width and depth of networks *ρ* and ϕ (both ϕ networks were fixed to be identical in size), dropout ratio, loss weight parameter *β*, block index embedding dimension *z* and learning rate. For the search space of hyperparmeters and example set of hyper parameters (see Table 1). Note that the dimension of the first layer of network *ρ* is determined by the dimension of the last layer of the networks $\phi_{C,E}$ and the size of the block embedding *z*. The layer dimensions for *ρ* in Table 1 are the layers following that first determined layer.

**Table 1.** Searched hyperparamater space (middle column), optimized hyperparameters for first fold using AML dataset (Fig. 3a) (right column).

Hyperparameter	Search space	Sample set
$\phi_{C},\;\phi_{E}$ networks dimensions	[[128,64], [64, 32, 16], [64, 32], [128, 64, 32]]	[128,64]
*ρ* network dimensions	[[128, 64], [64, 32, 16], [64, 32], [32, 16, 8], [32, 16]]	[64, 32]
Dropout ratio	[0, 0.2, 0.4, 0.7]	0
*β*	[0.25, 0.5, 0.75, 0.9, 0.99]	0.5
Block embedding size	[4, 8, 12, 16, 20]	8
Learning rate	[5e−5, 1e−4, 5e−4, 1e−3, 5e−3]	1e−3

The training process was conducted as follows: We split our orientation data for three disjoint groups of train, validation and test. For each evaluation, we fit three differently initialized models (all of them with the same optimized hyperparameter configuration), then choosing the one which performed the best over the validation set. This model was used for final evaluation over the test set. Each model was trained until a stopping condition of five consecutive epochs without improvement was met. We used ADAM for weight optimization (Kingma and Ba 2014). For the 5-fold cross validation, we repeated the process above five times (including the hyperparameter search).

We compared our approach to two previous ones: D2D (Silverbush and Sharan 2019) and that of Vinayagam *et al.* (2011). For D2D, we used the implementation in Silverbush and Sharan (2019) but incorporated it in our own framework. We reimplemented the method of Vinayagam *et al.* as portrayed in the original paper. This implementation is also available in our open GitHub repository. We could not use our own cause–effect dataset for this method due to low coverage of nodes by shortest paths. Hence, we used transcription factors and membrane receptors datasets as used in the original paper. Transcription factors were taken from Lambert *et al.* (2018) and Vaquerizas *et al.* (2009) and membrane receptors from Almén *et al.* (2009).

### 2.4 Data

**Cause–effect data.** Cancer genomic data were taken from TCGA spanning breast cancer, colon cancer, ovarian cancer and AML cancer patients. Following Silverbush and Sharan (2019) we defined the set of causal genes as those that were called mutated or had a copy number variation. The set of effect genes comprised all genes whose expression had an absolute fold change *z*-score > 3. We filtered out set pairs if one of the sets had >1000 genes. We also evaluated over drug response data taken from Silverbush and Sharan (2019). Guiding source dataset information is summarized in Table 2.

**Table 2.** Details of guiding sets used.

Guiding source	No. of guiding sets	Average set size	
		Cause	Effect
AML	205	110.4	298.6
Breast cancer	805	290.8	331.1
Colon cancer	419	336.2	375.3
Ovarian Cancer	219	263.8	460.9
Drug response	480	3.6	36.7

**Benchmark sets.** For training and validation, we used five sets of interactions with known directions. As negative samples, we used the opposite directions of these interactions. To prevent any degree bias, similar to Silverbush and Sharan (2019), we forced an equal number of interactions directed from high degree nodes to low degree nodes and vice versa. Interactions that had conflicting orientations in two different datasets were treated as undirected. We denote below the number of interactions in each of the datasets followed by the number of interaction after preprocessing inside parentheses.

1. 450 (432) signal-transduction interactions in mammalian cells (STKE) from Vinayagam *et al.* (2011).
2. 117 (110) interactions of the EGFR signaling pathway (EGFR) from Samaga *et al.* (2009).
3. 5762 (2510) kinase/phosphatase to substrate interactions (KPIs) from Phosphositeplus (Hornbeck *et al.* 2004).
4. 28 564 (198) protein–DNA interactions (PDIs) downloaded from ChEA (Lachmann *et al.* 2010).
5. 330 (326) E3 ubiquitination interactions, downloaded from hUbiquitome (Du *et al.* 2011).

For validation, we additionally used an independent set of 9176 directed interactions from Pathlinker (Ritz *et al.* 2016) that do not appear in the benchmark sets above.

**Cancer driver genes.** We assembled a list of 943 cancer driver genes coming from two sources:

1. Cancer Gene Census v95 (729 genes).
2. Following Hofree *et al.* (2016), we queried UniportKB (The UniProt Consortium 2016) for the keywords “proto-oncogene,” “oncogene,” and “tumor-suppressor gene” (417 genes).

We merged these lists and filtered out genes that did not appear in the PPI network.

**Disease genes.** The disease gene list compiled by Menche *et al.* (2015) contains genes associated with 299 diseases defined by the Medical Subject Headings (Mesh) (Lipscomb 2000) taken from the Online Mendelian Inheritance in Man(OMIM) (Kuhn *et al.* 2013) and genome-wide association study (GWAS) (Ramos *et al.* 2013) databases. Differentially expressed genes for these diseases were taken from the manual disease signature dataset in Creeds (Wang *et al.* 2016). We mapped diseases from both resources to their matching MeSH IDs to form gene set pairs. For each disease gene set we included all genes that were listed under descendent diseases in the MeSH tree. The resulting filtered set of diseases (after the mapping) included 207 diseases with an average of 115 causal genes and 1619 differentially expressed genes per disease.

**PPI network.** We used the weighted human PPI network from ANAT (Signorini *et al.* 2021). At the time of download, the network contained 483 206 interactions and 18 880 unique proteins. The network edges are weighted according to the reliability of the interactions based on the experimental techniques they were discovered.

## 3 Results

We designed a network orientation approach that starts from a collection of pairs of cause and effect sets and uses those sets to generate classification features for edge direction (Fig. 1). Our main source for cause–effect information is genomic data deposited in TCGA on mutated and differentially expressed genes in cancer patients. Each patient induces a set of mutated genes or genes with a copy number variation that are viewed as *causes*, as well as a set of differentially expressed genes (w.r.t. a normal tissue) that are viewed as *effects*. Information from these sets is diffused and then processed by a deep learning framework to generate features used for an edge orientation prediction.

Our main contribution is the latter feature generation process in which individual proximity scores between pairs of nodes are automatically aggregated and contrasted using the deep learning framework. This improves upon the previous D2D algorithm which uses sums and ratios to aggregate and contrast proximities. In more detail, for a set of causes *C*, a set of effects *E* and a potential orientation u→v of an edge, D2D computes a feature of the form $\mathrm{score}(u\to v)=\frac{F^{C}(u)\cdot F^{E}(v)}{F^{C}(v)\cdot F^{E}(u)}$, where $F^{C}(u)$ and $F^{E}(u)$ are the sum of proximity scores between *u* and nodes from cause and effect sets, respectively (and similarly for *v*). In contrast, D’or learns a general function on the set of proximities between *u* and *v* and cause and effect nodes to construct its directionality features.

### 3.1 Application and performance evaluation

We applied D’or to a network of unoriented PPIs in human with 483 206 interactions spanning 18 880 proteins (Signorini *et al.* 2021). As cause–effect data we used TCGA cancer genomic data from 1648 patients, where the causal set of each patient contained genes that had mutation or copy number variations and the effect set consisted of the differentially expressed genes of that patient (Methods). We benchmarked D’or using sets of interactions with known directions from five different sources: Kinase-substrate and phosphatase-substrate interactions (KPIs), protein–DNA interactions (PDIs), E3 ubiquitination interactions (E3), known directed interactions from the EGFR pathway (EGFR), and a collection of signaling interactions in mammalians from the signal transduction knowledge environment (STKE).

**Figure 3.**
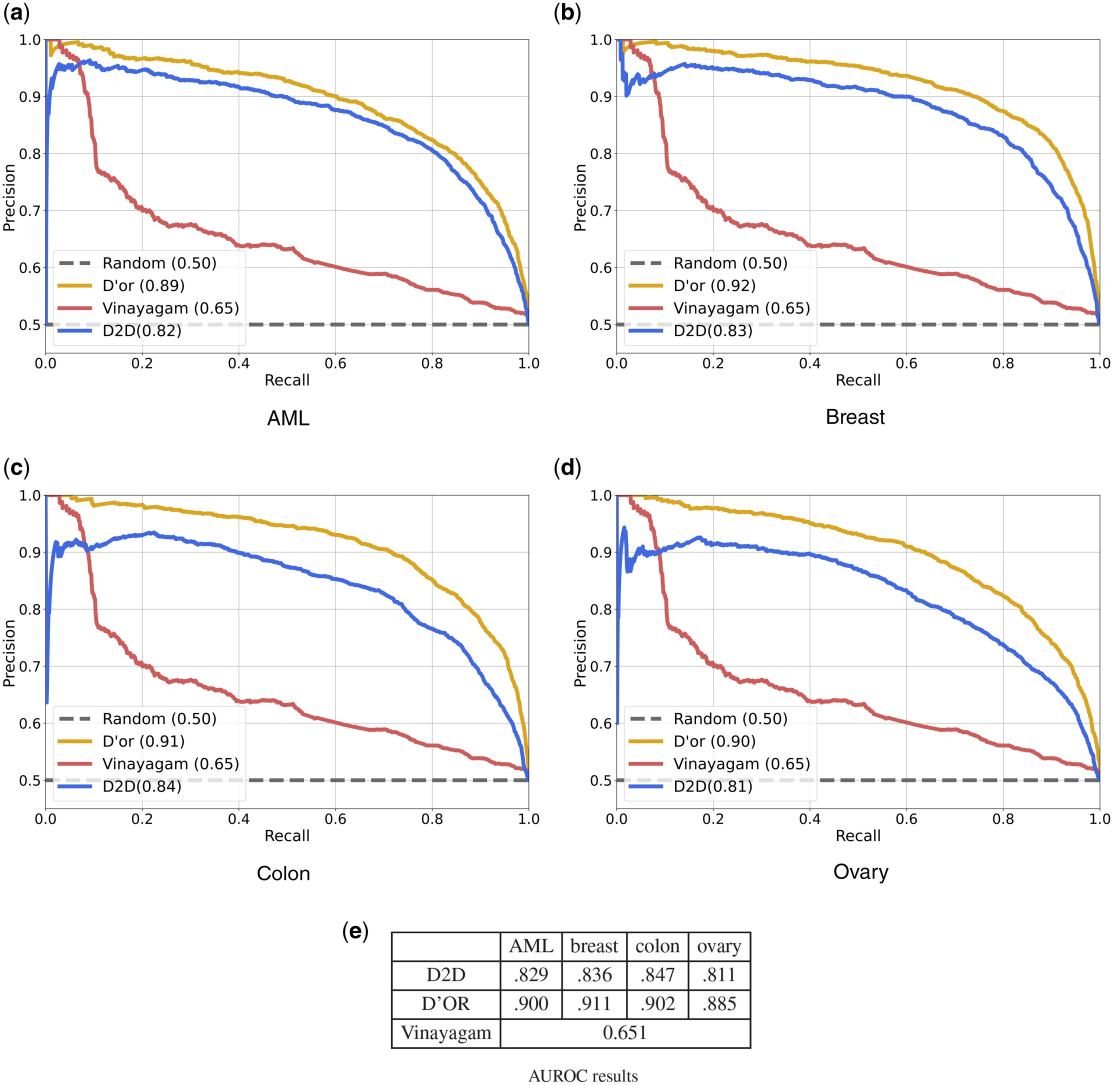
(a–d) Precision–recall curves using patient data from four different cancer types. (e) AUROC results for the same evaluations as in (a–d).

Performance evaluation was conducted using the area under the precision–recall curve (AUPRC) in a 5-fold cross validation test. D’or achieved an AUPRC of 0.89–0.92 compared to the previous state-of-the-art D2D with 0.81–0.86, and the method of Vinayagam *et al.* with 0.65 (Fig. 3). The results of the latter method are identical in all comparisons since we used their own features which do not depend on the patient data for evaluation (see Section 2). Similar results were obtained when using drug response cause–effect data (Supplementary Fig. S2).

**Figure 4.**
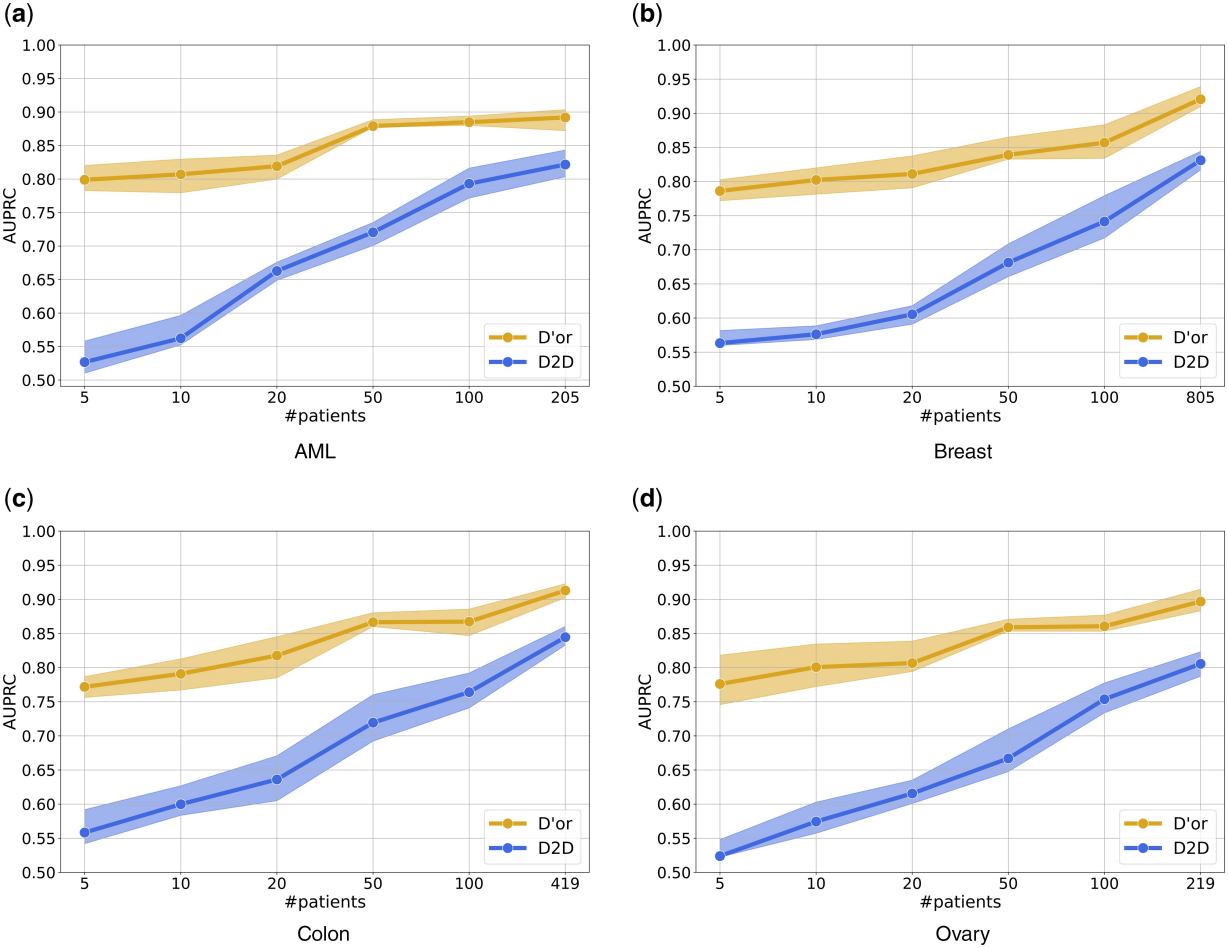
(a–d) Performance evaluation of D’or on an increasing number of patients of various cancer types. Shaded areas represent result boundaries over five different folds.

We further tested the performance as a function of number of patients used for feature generation. Figure 4 depicts prediction performance with an increasing number of patients. Evidently, D’or requires substantially less data than the other methods to achieve better results.

### 3.2 Consensus orientation

**Figure 5.**
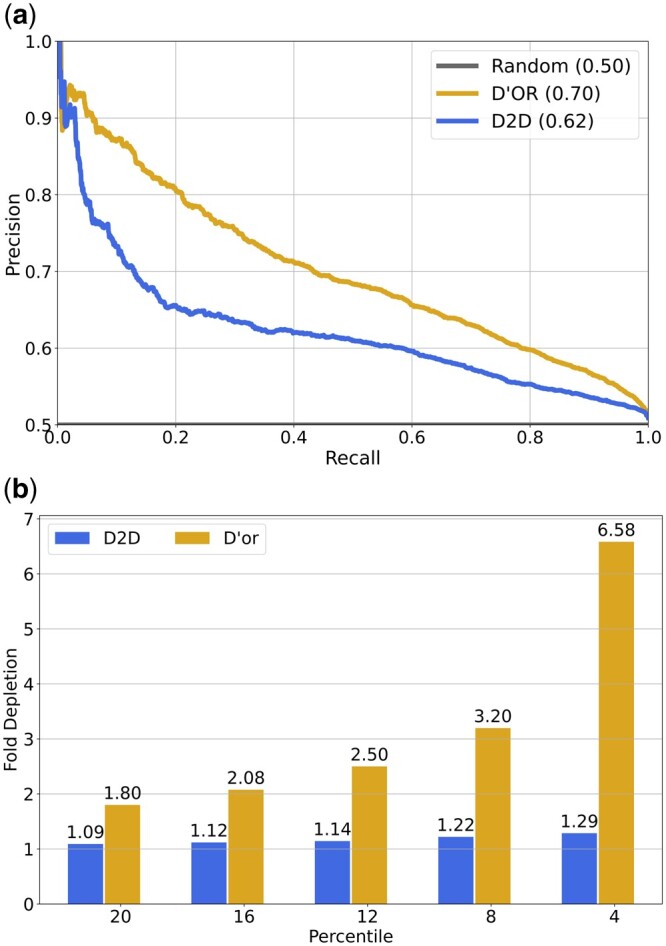
(a) AUPRC performance on an independent set of directed interactions from PathLinker. (b) Fold depletion of protein complex edges in top *K* percentile. Fold depletion was calculated as the percentage of protein complex edges in the whole network divided by the percent of protein complex edges found in the top *K* percentile.

After establishing the accuracy of our model, we turn to integrate the results from the four datasets into a single final score based on a log-likelihood score which takes into account each of the sources confidence of orientation (see Section 2 and Supplementary Fig. S2). We evaluated D’or consensus predictions on an independent set of directed interactions from the PathLinker database (Ritz *et al.* 2016). PathLinker comprises samples acquired through varied methods encompassing diverse interaction types. Demonstrating superior performance on an unfamiliar dataset illustrates that the generalizability of D’OR learning extends beyond the confines of its original training dataset, albeit with a corresponding decrease in performance (Fig. 5a). To further validate our predictions, we examined PPIs that reside within known protein complexes, with the assumption that such interactions should be left (to a large extent) unoriented by any orientation method. Indeed, we observe that such edges are significantly under represented in the group of edges with high confidence orientations (Fig. 5b).

To examine the functional role of proteins that are involved in interactions that were directed with high confidence, we focused on the 1% top scoring ones. We ranked the proteins by the number of such interactions they touch and subjected the 100 highest scoring proteins to GO slim enrichment analysis using the Panther tool v17.0 (Ashburner *et al.* 2000, Thomas *et al.* 2021, Aleksander *et al.* 2023). Expectedly, the most enriched terms (FDR corrected *p *<* *1E−12) include phosphorylation, regulation, signal transduction and protein modification (Table 3).

**Table 3.** GO enrichment analysis for proteins involved in the most confidently directed interactions.

GO description	GO ID	FDR
Phosphorylation	GO:0016310	6.01E−23
Protein phosphorylation	GO:0006468	6.4E−23
Regulation of cellular process	GO:0050794	2.37E−16
Phosphorus metabolic process	GO:0006793	7.29E−16
Phosphate-containing compound metabolic process	GO:0006796	7.89E−16
Regulation of biological process	GO:0050789	1.24E−15
Biological regulation	GO:0065007	8.98E−14
Signal transduction	GO:0007165	1.44E−13
Protein modification process	GO:0036211	2.18E−13
Cellular protein modification process	GO:0006464	2.43E−13

### 3.3 Gene prioritization

**Figure 6.**
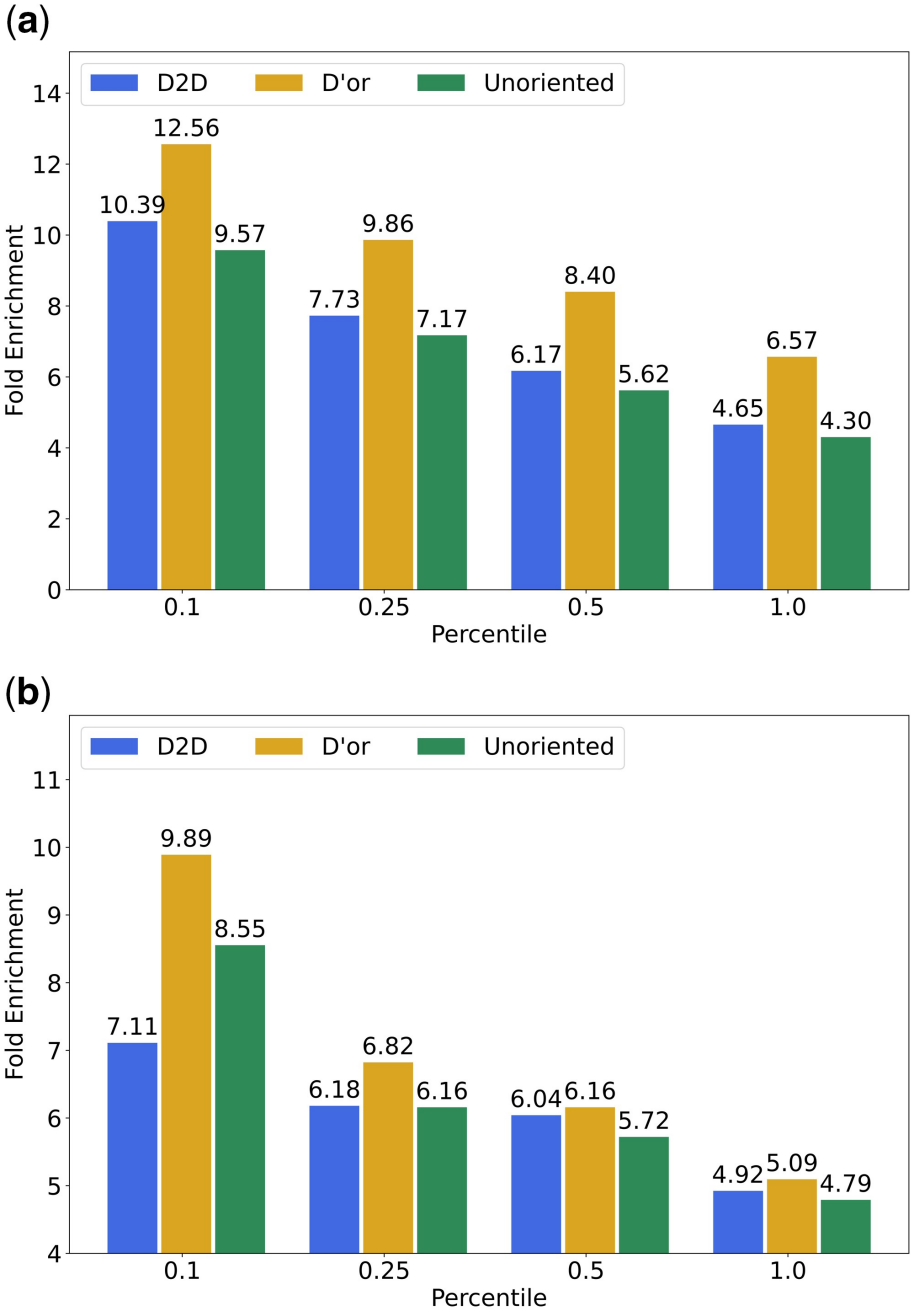
Prioritization performance. (a) Fold enrichment of cancer driver genes in the top *K* ranking percentile using AML differentially expressed genes. (b) Fold enrichment of disease-associated genes in the top *K* ranking percentile using differentially expressed genes of various diseases.

An oriented network facilitates the prioritization of disease-associated genes as it greatly reduces the space of (directed) paths that have to be explored (Silverbush and Sharan 2019). To assess the utility of the consensus network in gene prioritization, we conducted a leave-one-out test where each time we used three of the four patient datasets for direction prediction and aimed to predict cancer driver genes using the left out dataset. Since mutated genes may be directly informative of driver genes, we focused on prediction using differentially expressed genes only. To this end, we flipped edge directions (since we are interested in their upstream cause) and applied network propagation to the set of differentially expressed genes of each patient in the left-out set. We ranked genes for each patient separately and then aggregated the ranks and examined what portion of genes with the top *K* rank percentile (for varying *K* values) are known to be cancer driver genes. The focus of this experiment is to validate the accuracy of the algorithm’s orientation predictions. For that reason, we do not compare our results to other prioritization techniques. The prioritization results with respect to the AML dataset are given in Fig. 6a. Evidently, D’or outperforms D2D and an unoriented network across all *K* values. Similar results for the other cancer types appear in Supplementary Fig. S3.

As another test of the consensus orientation, we examined its utility in prioritizing genes associated with various diseases (see Methods), using all four datasets. Also in this test D’or outperformed D2D and an unoriented network across all *K* values (Fig. 6b).

## 4 Conclusions

We developed a network orientation method that is based on learning functions of sets of diffusion scores for pairs of cause and effect disease genes. Unlike previous approaches, D’or does not rely on heuristics and manually engineered features. Consequently, we presented a considerable improvement in orientation prediction over all tested scenarios. We further showed the utility of the oriented network in prioritizing cancer driver genes and other disease-related genes.

The framework we have presented can be extended in several ways. First, D’or only uses topological network information to make its predictions, even though its deep learning framework can easily incorporate additional information such as protein functional annotation. Second, while D’or provides limited explainability over its decision process, adopting explainable set learning methods (Hirsch and Gilad-Bachrach 2021) might shed more light on the underlying orientation mechanisms.

It is important to acknowledge that the utilization of four specific cancer-related datasets in this study might potentially restrict the generalization of the predictions to other conditions. Furthermore, while our scoring scheme ranks the interactions, it remains for future work to devise ways to learn a threshold that would distinguish the set of directed interactions from interactions that are likely to be undirected.

## Supplementary Material

btae355_Supplementary_Data
