# Supplementary material for: D’or: deep orienter of protein–protein interaction networks
Source: Bioinformatics. 2024 Jun 11;40(7):btae355. doi: 10.1093/bioinformatics/btae355 (PMC11254290; doi:10.1093/bioinformatics/btae355)
Supplement: btae355_Supplementary_Data [file btae355_supplementary_data.pdf]

Supplementary Information for:

D'or: Deep orienter of protein-protein interaction networks

Daniel Pirak[1] and Roded Sharan[2]

[1]School of Electrical Engineering, Tel Aviv University, Tel Aviv, 69978, Israel
[2]School of Computer Science, Tel Aviv University, Tel Aviv, 69978, Israel

A Supplementary figures

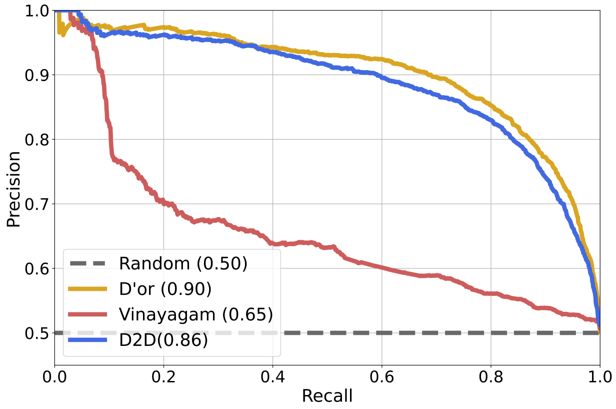

Figure S1: Performance of D'or using drug response data from Silverbush and Sharan (2019).

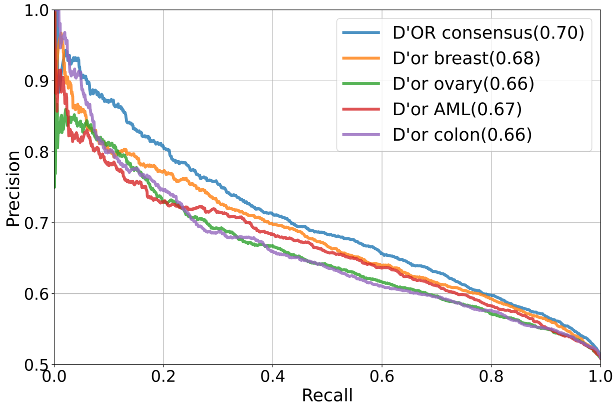

Figure S2: Performance of D'or consensus score versus each cancer type separately on the PathLinker test set.

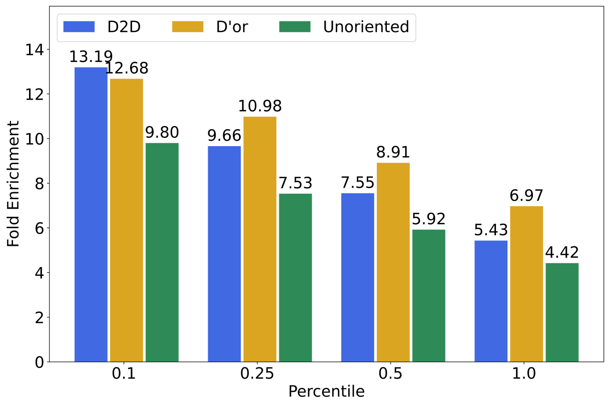

(a) Breast

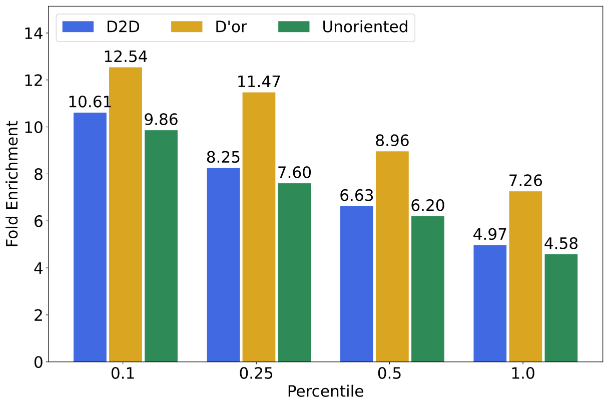

(b) Colon

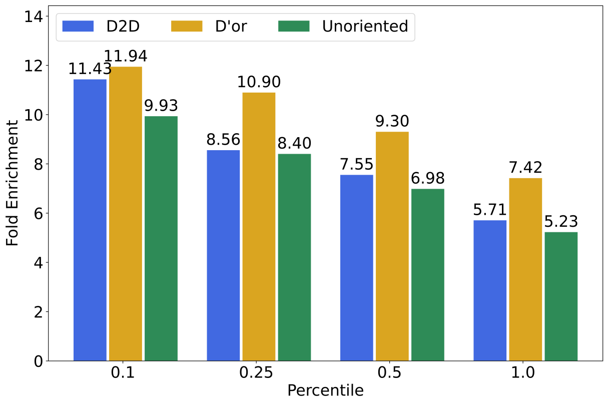

(c) Ovary

Figure S3: Prioritization performance. (a-c) Fold enrichment of cancer driver genes in the top K ranking percentile using differentially expressed genes of various cancer types.

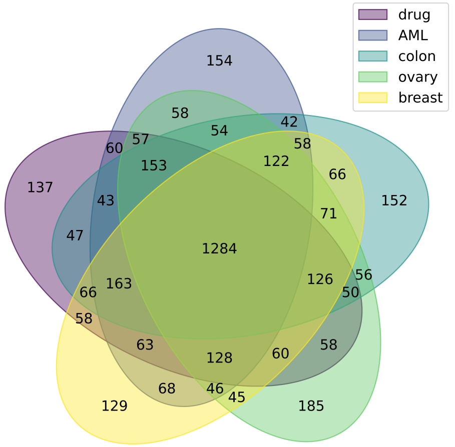

Figure S4: Orientation agreement of different cause-effect datasets over our entire orientation dataset, determined through a 5-fold cross-validation. For cancer, we employed genetic perturbations and their effects on differentially expressed genes to orient the edges in the network, while for drug dataset, we used the relationship between drug targets and differentially expressed genes.

References

Silverbush, D. and Sharan, R. (2019). A systematic approach to orient the human protein–protein interaction network. *Nature Communications*, **10**(1).
